# Analysis and research on clinical factors and treatment of infection in oral and maxillofacial space infection

**DOI:** 10.1186/s12903-025-06885-4

**Published:** 2025-09-29

**Authors:** Xuan Zhang, Chao Li, Rui Wang, Rui Liu, Hongjuan Lv

**Affiliations:** Department of Stomatology, The First Affiliated Hospital of Hebei North University, No.12 Changqing Road, Qiaoxi District, Zhangjiakou City, Hebei Province 075000 China

**Keywords:** Oral and maxillofacial space infection, Clinical factors, Treatment, Support vector machine

## Abstract

**Background:**

Oral and maxillofacial space infection (OMSI) is a relatively severe type of infection in the oral and maxillofacial region, typically involving the interstitial soft tissues of the face and neck. There is still a significant gap in the systematic analysis of clinical factors in existing studies. This study aims to explore the clinical factors influencing Oral and Maxillofacial Space Infection and analyze their relevance to treatment.

**Methods:**

A total of 408 cases of patients with oral and maxillofacial space infection were collected from the department of Oral and Maxillofacial Surgery of the First Hospital Affiliated to North China University of Science and Technology. Clinical data were classified, recorded, and statistically analyzed. Pearson’s chi-square test was used to preliminarily screen the independent variables, excluding irrelevant factors; Spearman correlation was used to calculate the correlation between independent and dependent variables; Logistic regression analysis was further conducted to explore factors with more significant risk. Support vector machine analysis was performed.

**Results:**

Pearson’s chi-square test and Spearman correlation analysis showed that gender, age, length of hospital stay, trismus, dysphagia, odontogenic origin, and surgery were associated with the degree of oral and maxillofacial space infection. Single-factor logistic regression analysis showed that gender (OR = 1.834, 95% CI: 1.211–2.755, *P* = 0.004), age (OR = 0.584, 95% CI: 0.384–0.889, *P* = 0.012), length of hospital stay (OR = 0.649, 95% CI: 0.426–0.990, *P* = 0.045), trismus (OR = 5.448, 95% CI: 3.252–9.127, *P* < 0.001), dysphagia (OR = 31.590, 95% CI: 7.637-130.665, *P* < 0.001), odontogenic origin (OR = 2.340, 95% CI: 1.267–4.323, *P* = 0.007), and surgery (OR = 2.847, 95% CI: 1.869–4.337, *P* < 0.001) were associated with oral and maxillofacial space infection. Multifactor logistic regression showed that gender (OR = 2.006, 95% CI: 1.214–3.315, *P* = 0.007), age (OR = 0.503, 95% CI: 0.301–0.841, *P* = 0.009), trismus (OR = 4.225, 95% CI: 2.337–7.640, *P* < 0.001), dysphagia (OR = 31.071, 95% CI: 7.062-136.709, *P* < 0.001), and surgery (OR = 2.653, 95% CI: 1.620–4.347, *P* < 0.001) were associated with oral and maxillofacial space infection. Support vector machine (SVM) analysis revealed a significant correlation between OMSI and length of hospital stay and age.

**Conclusion:**

The severity of oral and maxillofacial space infection is related to age, trismus, dysphagia, surgery, and length of hospital stay, which may influence the occurrence, development, and treatment outcomes of oral and maxillofacial space infection. It provides an evidence-based reference for clinical treatment decisions, which has important clinical application value and public health significance.

## Introduction

Oral maxillofacial space infection (OMSI) is a severe and potentially life-threatening condition characterized by the rapid spread of infection through anatomical spaces in the head, face, and neck, which may lead to complications such as airway obstruction, sepsis, and cavernous sinus thrombosis[1, 2]. OMSI can significantly impair patients’ daily activities and overall health, and delayed or ineffective treatment is associated with high mortality[3]. Epidemiological studies indicate that most OMSI cases are odontogenic in origin, with some reports attributing up to 83% of cases to odontogenic infections[4]. However, clinical progression is also influenced by factors such as the source of infection, timing of treatment, and patient immune status. Although less common, non-odontogenic causes (e.g., trauma, upper respiratory tract infections) may also result in OMSI and warrant further investigation[5, 6].

Current management of OMSI emphasizes timely diagnosis, effective infection control, and multidisciplinary collaboration, particularly between dentists and otolaryngologists in complex cases[7]. While traditional statistical methods are widely used to analyze clinical risk factors, they may have limited ability to capture complex nonlinear interactions among variables. Machine learning techniques, such as Support Vector Machine (SVM), offer an alternative approach with enhanced capacity to identify subtle patterns in high-dimensional data. SVM has been successfully applied in various medical fields, including neonatal seizure detection, sepsis mortality prediction, and oral pathology[8–10], highlighting its potential in predicting disease severity and supporting clinical decision-making.

These applications highlight the potential of SVM in predicting disease severity and guiding treatment decisions. Therefore, this study aims to identify clinical factors associated with OMSI severity and treatment outcomes, and to evaluate the utility of SVM in capturing nonlinear relationships among these factors alongside traditional statistical methods.

## Methods

### Case selection

A total of 408 cases of patients with oral and maxillofacial space infection admitted to the Department of Oral and Maxillofacial Surgery, the First Hospital Affiliated to North China University of Science and Technology, from September 2012 to September 2022 were collected. Mild infection is generally confined to a single space, local symptoms are relatively mild, and systemic reactions are not obvious. Moderate infection may involve multiple adjacent Spaces, local swelling and pain are more obvious, and may be accompanied by mild systemic symptoms such as low-grade fever and fatigue. Severe infection can extensively involve multiple Spaces, which may affect important functions such as breathing and swallowing. The systemic symptoms are serious, such as high fever, chills, sepsis, and even life-threatening. This study was approved by the hospital’s ethics committee, and all patients provided written informed consent. This study has been approved by the hospital ethics committee (ethics number: [K2021279]) and all patients provided written informed consent.

- Inclusion criteria: (1) Patients with a confirmed diagnosis of OMSI and treated with intravenous antibiotics. (2) Patients who were hospitalized and received intravenous antibiotics for oral and maxillofacial space infections at our hospital. (3) Age ≥ 18 years old. (4) Relevant medical records were complete. (5) Relevant oral radiographic and ultrasound data were available. (6) Relevant microbiological examination data and antibiotic sensitivity test data were complete.
- Exclusion criteria: (1) Patients with immune system dysfunction. (2) Patients with severe acute infections requiring immediate intervention (e.g., septic shock, multi-organ failure) or those who have not received appropriate treatment prior to admission. (3) Patients who could not understand the research content or provide informed consent.

Note We acknowledge the potential selection bias due to the inclusion of only hospitalized patients. This limitation has been clearly stated in the methods section, and we acknowledge that the findings may not be fully generalizable to all OMSI patients, particularly those who receive outpatient treatment.

### Detailed statistical analysis of case information

According to the clinical data of patients, classification was performed based on gender (male/female), age (< 60/≥60 years), length of hospital stay (< 10/≥10 days), trismus (yes/no), dysphagia (yes/no), odontogenic etiology (yes/no), diabetes (yes/no), and surgery (yes/no). Among them, the “surgical” variable refers to whether the patient has undergone any surgical intervention related to the treatment of oral maxillofacial space infection. Specifically, it includes but is not limited to the following types of surgery:

- Abscess drainage: Incision drainage of the formed abscess to reduce local pressure and infection.
- Debridement: Debridement of infected tissue to remove necrotic tissue and promote healing.
- Other related surgeries: such as local tissue repair, osteotomy, etc., to treat tissue damage or complications caused by infection.

All surgical interventions described above were performed in hospitalized patients included in this study.

### Support vector machine (SVM)

Support Vector Machine (SVM) is a machine learning algorithm mainly used for classification and regression analysis. Its basic idea is to find an optimal hyperplane in a high-dimensional space to separate data points of different categories, maximizing the margin between two categories. This hyperplane is called the decision boundary, and the data points closest to this boundary are called support vectors. SVM is widely used in classification problems, especially in handling high-dimensional data and linearly inseparable cases. In addition to linear classification, SVM can also perform nonlinear classification through kernel functions, mapping data to high-dimensional space to make it linearly separable.

Justification for SVM Use: Although traditional regression models are commonly used in medical research, SVM was chosen for its ability to model nonlinear relationships between clinical factors and OMSI severity. This method is particularly useful when dealing with complex interactions between multiple variables that may not be captured by linear regression models. SVM provides a more accurate classification, especially in high-dimensional datasets, making it a suitable choice for this study.

Model development and evaluation:

- Kernel function: The radial basis function (RBF) kernel was selected because of its flexibility in handling nonlinear decision boundaries.
- Data preprocessing: Continuous variables were standardized to zero mean and unit variance before model fitting to ensure that all features were on the same scale.
- Parameter tuning: The penalty parameter (C) and kernel coefficient (γ) were optimized using a grid search method within a 10-fold cross-validation on the training set.
- Data splitting: The dataset was randomly divided into a training set (70%) and a testing set (30%).
- Performance evaluation: Model accuracy, sensitivity, specificity, and the area under the receiver operating characteristic curve (AUC) were calculated on the independent test set.
- Software and reproducibility: All analyses were conducted using Python (scikit-learn Library, version 1.2.0) with a fixed random seed to ensure reproducibility.

The clinical data of oral and maxillofacial space infection often have the problems of limited sample size and high data dimension. SVM is especially suitable for classification and regression of small samples and high-dimensional data. It can find the optimal classification hyperplane or regression model under limited data to avoid overfitting. SVM maps low-dimensional nonlinear data into high-dimensional space by kernel function, which can effectively deal with the nonlinear relationship in the data and better explore the complex internal relationship between the clinical factors of oral and maxillofacial space infection and treatment outcomes. The SVM model was trained and tested for many times to ensure that the performance of the model was stable and reliable, and to avoid the bias caused by the contingency of data partitioning. At the same time, the performance of the model on different data subsets was analyzed to verify that SVM could maintain good prediction performance and provide superior performance when processing the clinical data of oral and maxillofacial space infection. First use cross-validation for model selection and hyperparameter tuning, then use a separate test set for the final model evaluation to obtain more reliable results.

### Statistical methods

The clinical data collected were organized using EXCEL software database, and the clinically organized data were analyzed statistically using the SPSS software package. Data were represented as percentages of the total. Pearson’s chi-square test was used to preliminarily screen independent variables, excluding irrelevant factors; Spearman correlation was used to calculate the correlation between independent and dependent variables; Logistic regression analysis was further conducted to explore factors with more significant risk. The Variance Inflation Factor (VIF) was used to assess multicollinearity among independent variables, particularly between trismus and dysphagia, ensuring that no significant collinearity issues affected the regression results. A p-value < 0.05 indicated significant differences with statistical significance.

## Results

### Pearson chi-square test analysis of factors associated with oral and maxillofacial space infection

The Pearson chi-square test summarized the factors associated with oral and maxillofacial space infection. In individuals, gender (*P* = 0.004), age (*P* = 0.012), length of hospital stay (*P* = 0.044), trismus (*P* < 0.001), dysphagia (*P* < 0.001), odontogenic origin (*P* = 0.006), and surgery (*P* < 0.001) were correlated with the severity of oral and maxillofacial space infection. Diabetes (*P* = 0.637) showed no correlation with the severity of infection (Table 1).

**Table 1:** Pearson chi-square test to analyze factors associated with oral and maxillofacial space infection

Parameters		Oral and maxillofacial space infection		P
		Yes	No	
Gender				0.004*
Male	247	174 (42.6%)	73(17.9%)	
Female	161	91 (22.3%)	70 (17.2%)	
Age				0.012*
<60 years	231	138 (33.8%)	93 (22.8%)	
≥ 60 years	177	127 (31.1%)	50 (12.3%)	
Length of stay				0.044*
<10 days	241	147 (36.0%)	94 (23.0%)	
≥ 10 days	167	118 (28.9%)	49 (12.0%)	
Trismus				< 0.001*
Yes	324	237 (58.1%)	87 (21.3%)	
No	84	28 (6.9%)	56 (13.7%)	
Dysphagia				< 0.001*
Yes	84	82 (20.1%)	2 (0.5%)	
No	324	183 (44.9%)	141 (34.6%)	
Odontogenic				0.006*
Yes	361	243 (59.6%)	118 (28.9%)	
No	47	22 (5.4%)	25 (6.1%)	
Diabetes				0.637
Yes	91	61 (15.0%)	30 (7.4%)	
No	317	204 (50.0%)	113 (27.7%)	
Surgery				< 0.001*
Yes	219	166 (40.7%)	53 (13.0%)	
No	189	99 (24.3%)	90 (22.1%)	

*P < 0.05

### Spearman correlation test further analyzing factors associated with oral and maxillofacial space infection

To confirm factors associated with oral and maxillofacial space infection, further Spearman correlation analysis was conducted. Gender (ρ = 0.143, *P* = 0.004), age (ρ = −0.125, *P* = 0.012), length of hospital stay (ρ = −0.100, *P* = 0.044), trismus (ρ = 0.337, *P* < 0.001), dysphagia (ρ = 0.349, *P* < 0.001), odontogenic origin (ρ = 0.137, *P* = 0.006), and surgery (ρ = 0.245, *P* < 0.001) were correlated with oral and maxillofacial space infection. Diabetes (ρ = 0.023, *P* = 0.638) showed no correlation with oral and maxillofacial space infection (Table 2).

**Table 2:** Spearman correlation analysis of factors associated with oral and maxillofacial space infection

Parameters	Oral and maxillofacial space infection	
	ρ	*P*
Gender	0.143	0.004*
Age	−0.125	0.012*
Length of stay	−0.100	0.044*
Trismus	0.337	< 0.001*
Dysphagia	0.349	< 0.001*
Odontogenic	0.137	0.006*
Diabetes	0.023	0.638
Surgery	0.245	< 0.001*

*P < 0.05

### Single-factor logistic regression analysis of oral and maxillofacial space infection and related factors

Single-factor logistic regression was used to understand and quantify the impact of related factors on oral and maxillofacial space infection. Table 3. describes the odds ratios (OR) and 95% confidence intervals (CI) of the study subjects using single-factor logistic regression. Gender (OR = 1.834, 95% CI: 1.211–2.755, *P* = 0.004), age (OR = 0.584, 95% CI: 0.384–0.889, *P* = 0.012), length of hospital stay (OR = 0.649, 95% CI: 0.426–0.990, *P* = 0.045), trismus (OR = 5.448, 95% CI: 3.252–9.127, *P* < 0.001), dysphagia (OR = 31.590, 95% CI: 7.637-130.665, *P* < 0.001), odontogenic origin (OR = 2.340, 95% CI: 1.267–4.323, *P* = 0.007), and surgery (OR = 2.847, 95% CI: 1.869–4.337, *P* < 0.001) were associated with oral and maxillofacial space infection. Diabetes (OR = 1.126, 95% CI: 0.687–1.846, *P* = 0.637) showed no correlation with oral and maxillofacial space infection (Table 3.).

**Table 3.** Univariate Logistic regression analysis of factors associated with oral and maxillofacial space infection

Parameters	Oral and maxillofacial space infection		
	OR	95%CI	P
Gender			0.004*
Male	1		
Female	1.834	1.211–2.755	
Age			0.012*
<60 years	1		
≥ 60 years	0.584	0.384–0.889	
Length of stay			0.045*
<10 days	1		
≥ 10 days	0.649	0.426–0.990	
Trismus			< 0.001*
Yes	1		
No	5.448	3.252–9.127	
Dysphagia			< 0.001*
Yes	1		
No	31.590	7.637-130.665	
Odontogenic			0.007*
Yes	1		
No	2.340	1.267–4.323	
Diabetes			0.637
Yes	1		
No	1.126	0.687–1.846	
Surgery			< 0.001*
Yes	1		
No	2.847	1.869–4.337	

*P < 0.05

### Multi-factor logistic regression analysis of factors associated with oral and maxillofacial space infection

Multi-factor logistic regression simultaneously considers the impact of multiple factors on oral and maxillofacial space infection. Multi-factor logistic regression analysis was used to study the ORs and 95% CIs of the study subjects at the multivariable level. Multi-factor logistic regression showed that gender (OR = 2.006, 95% CI: 1.214–3.315, *P* = 0.007), age (OR = 0.503, 95% CI: 0.301–0.841, *P* = 0.009), trismus (OR = 4.225, 95% CI: 2.337–7.640, *P* < 0.001), dysphagia (OR = 31.071, 95% CI: 7.062-136.709, *P* < 0.001), surgery (OR = 2.653, 95% CI: 1.620–4.347, *P* < 0.001) were associated with oral and maxillofacial space infection. Odontogenic origin (OR = 1.910, 95% CI: 0.891–4.094, *P* = 0.096) lost its statistical significance, which may suggest confounding effects from other clinical factors, such as trismus, dysphagia, and the necessity for surgical intervention. These factors were strong independent predictors in the multivariate model, and when controlling for them, the significance of odontogenic origin diminished. Diabetes (OR = 1.127, 95% CI: 0.612–2.076, *P* = 0.702) showed no correlation with oral and maxillofacial space infection (Table 4).

**Table 4:** Multivariate logistic regression analysis of factors associated with oral and maxillofacial space infection

Parameters	Oral and maxillofacial space infection		
	OR	95% CI	*P*
Gender	2.006	1.214–3.315	0.007*
Age	0.503	0.301–0.841	0.009*
Length of stay	0.862	0.515–1.445	0.573
Trismus	4.225	2.337–7.640	< 0.001*
Dysphagia	31.071	7.062-136.709	< 0.001*
Odontogenic	1.910	0.891–4.094	0.096
Diabetes	1.127	0.612–2.076	0.702
Surgery	2.653	1.620–4.347	< 0.001*

*P < 0.05

### Clinical factor analysis of oral and maxillofacial space infection based on support vector machine (SVM)

In this study, we used a support vector machine (SVM) model to analyze the clinical factors of oral maxillofacial space infection. The SVM model is used to predict the binary variable “infection” and is validated with 50% cross-validation to ensure that the proportion of categories in each compromise is the same as that of the entire dataset, thereby reducing bias. In the model implementation, we defined specific cut-off points: age less than 60 years (younger patients may have different immune responses and resilience based on the effect of age on infection severity) and hospital stay less than 10 days (because hospital stay is related to disease severity, and shorter hospital stay may mean milder disease).

SVM models are trained and tested through 50% cross-validation, with 70% of the data used for training and 30% for testing. Performance metrics include AUC (area under the curve), sensitivity, and specificity. The average training AUC was 0.808 and the average test AUC was 0.786. The mean training sensitivity was 0.742 and the mean test sensitivity was 0.815. The mean training specificity was 0.747 and the mean test specificity was 0.697. These results show that the model performs well on both the training and test sets, with high sensitivity and specificity.

In addition, we calculate other performance metrics, including accuracy, precision, and F1 scores. The average training accuracy was 0.750 and the average test accuracy was 0.730. The average training accuracy was 0.760 and the average test accuracy was 0.740. The average training F1 score is 0.751 and the average test F1 score is 0.776. These indicators further verify the stability and generalization ability of the model.

In terms of data partitioning and cross-validation procedures, we use 50% cross-validation to ensure that the proportion of each compromise category is the same as that of the entire dataset. Hyperparameter tuning uses grid search combined with cross-validation, and the final selected hyperparameters are C = 1.0 and γ = 0.1. The classification results are as follows: The confusion matrix shows that the true cases (TP) in the test set are 215, the false positive cases (FP) are 43, the true negative cases (TN) are 100, and the false negative cases (FN) are 50. These results show that the model performs well on both the training and test sets, with high sensitivity and specificity.

**Fig. 1:**
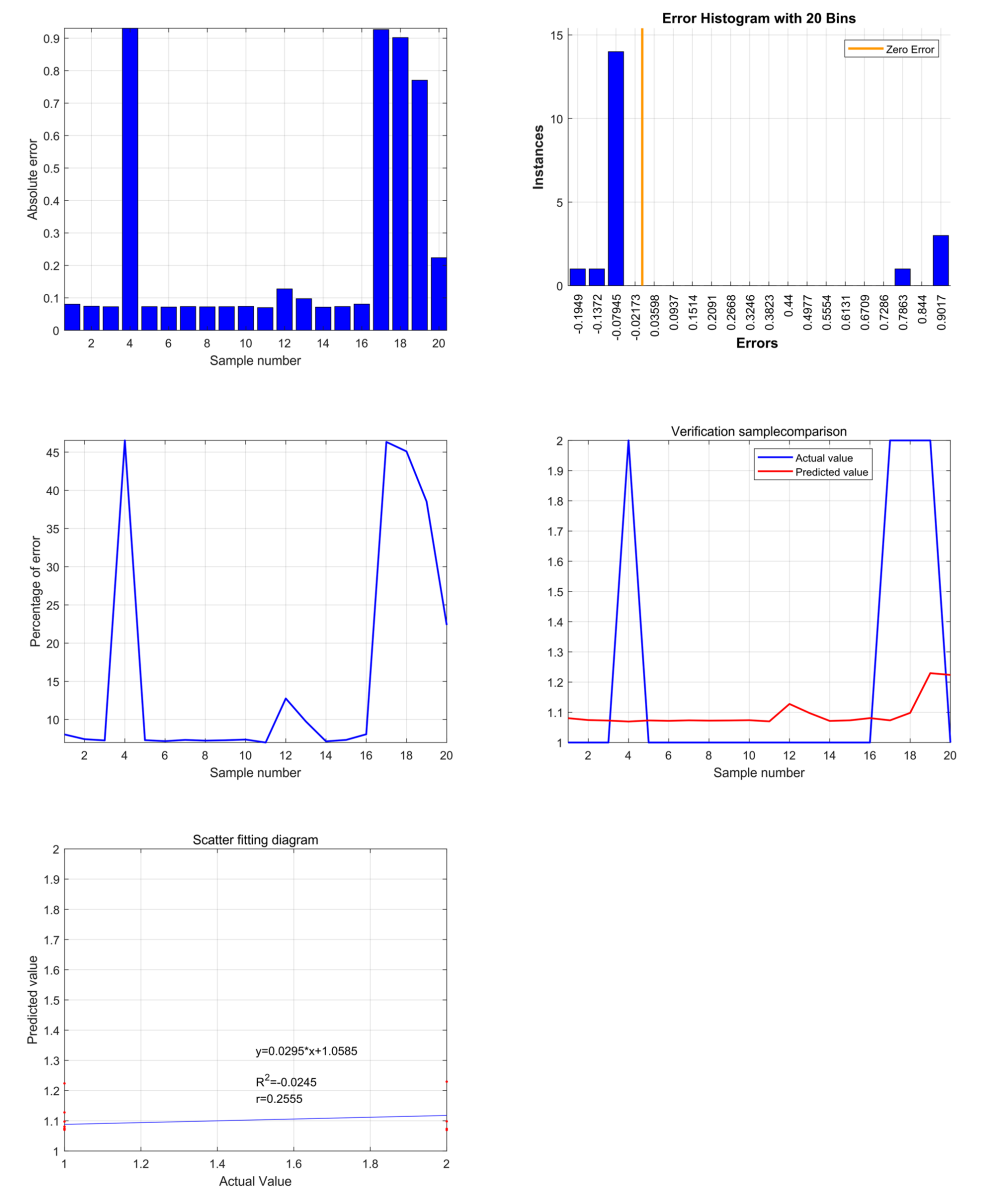
Clinical factor analysis of oral and maxillofacial space infection based on support vector machine (svm). there is a strong correlation between length of hospital stay and age with oral and maxillofacial space infection (y = 0.0295*x + 1.0585), with an error mean of 0.25%

The relationship between length of hospital and age and oromaxillofacial space infection can be described by the equation y = 0.0295 × 1.0585 with a mean error rate of 0.25% (Fig. 1). These findings provide valuable references for identifying and managing oral and maxillofacial space infections in clinical practice.

## Discussion

Oral and maxillofacial space infection (OMSI) is a severe condition that can rapidly spread through the anatomical spaces of the head, face, and neck, leading to potentially fatal complications such as sepsis and airway obstruction [11]. However, while the rapid spread of OMSI is widely acknowledged, it is critical to recognize that the severity of these complications varies depending on the infection’s source, the timeliness of treatment, and individual patient factors such as immune status. For example, trismus, difficulty swallowing, and respiratory distress, often resulting from local inflammation and tissue necrosis, can be alleviated with early antibiotic therapy and surgical intervention [12]. OMSI typically arises from dental infections, postoperative infections, or trauma, with odontogenic infections being the most common cause. However, the clinical progression of OMSI can differ greatly based on the infection source. While odontogenic infections are a leading cause of OMSI, some infections may be self-limiting, while others can progress rapidly to severe outcomes. This variability suggests that odontogenic infections may not always be as directly correlated with OMSI severity as traditionally believed, and it highlights the need for a more comprehensive assessment of other clinical factors [13, 14].

The relationship between age and the risk of OMSI is multifaceted, with both older adults and children being more vulnerable due to their immune system status and oral hygiene habits [15]. While aging is generally associated with weakened immunity and increased oral health issues, it is not an absolute predictor of OMSI severity. Similarly, children may be at risk due to developing immune systems and exposure to factors like poor oral hygiene and deciduous tooth shedding [16, 17]. However, age alone cannot fully explain the variation in disease severity. Comprehensive assessments, including immune function and lifestyle factors, are necessary to predict and manage OMSI risk effectively. Future research should prioritize age-specific interventions, especially for high-risk groups, to refine preventive measures and enhance clinical outcomes [18].

Trismus is a frequent complication of OMSI, often caused by inflammation and swelling in the maxillofacial region [19]. While effective antibiotic and anti-inflammatory treatment is essential for controlling the infection, managing trismus requires a more holistic approach that addresses both the underlying infection and the associated complications. The development of trismus highlights the importance of early detection, as it can lead to airway obstruction and significant functional impairment if left untreated. Future research should focus on optimizing treatment protocols that combine both systemic and local interventions to improve recovery outcomes for patients with OMSI-related trismus [20].

OMSI frequently involves dysphagia and dyspnea due to severe inflammation in the throat region, including the tonsils and posterior pharynx [21]. This inflammation may cause pain, swelling, and compromised swallowing function, which in severe cases may lead to throat spasms, difficulty breathing, and airway obstruction. Furthermore, the systemic effects of OMSI, including fever and fatigue, can worsen respiratory function. These complications underline the importance of early intervention to manage inflammation, alleviate symptoms, and prevent airway collapse. Timely medical attention is critical in mitigating respiratory distress and improving patient outcomes.

Surgical intervention is often required in severe OMSI cases, particularly when abscess formation occurs, increasing local pressure and causing pain and dysfunction [22]. Surgical drainage is essential to alleviate symptoms and prevent further spread of infection, particularly to surrounding tissues, blood vessels, and bones [23]. In untreated cases, OMSI may cause irreversible damage to tissues, necessitating surgical repair. Early diagnosis and timely intervention are critical to prevent complications and preserve function, underscoring the need for a comprehensive treatment approach.

The severity of OMSI directly impacts the length of hospital stay. Mild cases may require only short-term observation and antibiotic therapy, while severe cases necessitate prolonged hospitalization and possibly multiple surgical interventions [24]. Antibiotic therapy, particularly broad-spectrum β-lactams and cephalosporins, is the cornerstone of treatment, especially in less severe cases. These antibiotics provide effective coverage against common aerobic and anaerobic pathogens [25]. While surgical interventions are often necessary for managing severe OMSI, non-surgical treatments, such as antibiotic therapy alone, are commonly employed in less severe cases. A comparison of surgical vs. non-surgical treatment approaches would provide valuable insights into the most effective treatment strategies, especially for mild-to-moderate cases. Future studies should explore the effectiveness, safety, and cost-effectiveness of these two approaches, considering factors such as infection severity and patient comorbidities.

Anti-inflammatory treatment plays a crucial role in managing OMSI, particularly in controlling pain and inflammation. Nonsteroidal anti-inflammatory drugs (NSAIDs) like ibuprofen or diclofenac are frequently employed for their analgesic and anti-inflammatory properties. However, in severe cases with significant swelling or potential airway obstruction, corticosteroids such as dexamethasone are often used to manage excessive inflammation and facilitate healing. While corticosteroid therapy has been widely discussed in oral surgery, particularly for reducing post-operative inflammation and improving tissue recovery [26], its use must be balanced against potential side effects, particularly in immunocompromised patients. Future studies should aim to better define the indications for steroid use in OMSI to optimize clinical outcomes without compromising patient safety. If the treatment plan is complex, longer hospitalization may be required. The patient’s immune status also plays an important role in the treatment and recovery from infection. Patients with weakened immune function may require longer hospitalization to cope with the infection and may also require closer monitoring and care. OMSI may lead to various complications, such as septic shock, intracranial infection, etc., which may prolong hospitalization and require closer monitoring and treatment.

The findings of this study highlight key clinical indicators—trismus, dysphagia, and surgical intervention—as strong predictors of OMSI severity. The results show a significant association between these factors and severe outcomes (e.g., trismus OR = 4.225, dysphagia OR = 31.071), reinforcing the importance of early identification and timely intervention. However, while these indicators are valuable, their predictive power may vary depending on individual patient circumstances, such as the presence of other comorbidities or the timing of treatment. Thus, clinicians should not only focus on these variables in isolation but also incorporate a broader clinical assessment when determining management strategies. Second, the study highlights the importance of individualized treatment approaches. While broad-spectrum antibiotics remain the cornerstone of OMSI treatment, patients presenting with severe dysphagia or airway compromise may benefit from adjunct corticosteroid therapy to reduce inflammation and improve respiratory function. This is particularly relevant given that severe inflammation can exacerbate upper airway obstruction, necessitating early intervention. Third, the non-significant role of odontogenic origin in multivariate analysis suggests that clinicians should not rely solely on infection source as a predictor of severity. Instead, a comprehensive assessment considering multiple factors (e.g., immune status, hospital stay duration, presence of systemic disease) should be integrated into clinical workflows. By incorporating these findings into clinical practice, healthcare professionals can improve early detection, optimize treatment strategies, and potentially reduce hospital stays and complications associated with OMSI.

In the univariate logistic regression analysis, odontogenic origin was significantly associated with OMSI severity (OR = 2.340, *P* = 0.007), suggesting that infections originating from dental sources are more likely to result in severe outcomes. However, in the multivariate model, this association was no longer statistically significant (OR = 1.910, *P* = 0.096). This discrepancy may be attributed to confounding effects from other clinical factors, particularly trismus, dysphagia, and the necessity for surgical intervention. Many odontogenic infections progress to severe OMSI cases only when accompanied by these complications, which were found to be strong independent predictors of severity in the multivariate analysis (e.g., dysphagia: OR = 31.071, *P* < 0.001). When controlling for these factors, odontogenic origin itself may not directly contribute to severity but rather serves as an initial risk factor that, in combination with other variables, influences disease progression. Another possible explanation is that odontogenic infections vary in their clinical presentation and severity. Some odontogenic infections may be mild and self-limiting, while others can progress rapidly to life-threatening conditions. This heterogeneity may reduce the statistical significance of odontogenic origin as an independent predictor when multiple factors are accounted for. Therefore, while odontogenic infections are a primary source of OMSI, their impact on severity may be mediated by additional clinical variables, which must be considered in comprehensive risk assessments.

In addition to logistic regression, we employed SVM analysis to further explore the relationships between clinical factors and OMSI severity. While logistic regression is effective in identifying risk factors and quantifying their effects, it assumes linearity, which may not fully capture the complex interactions among variables. SVM, on the other hand, allows for nonlinear classification, enabling it to uncover patterns in the data that may not be apparent in traditional statistical models. For instance, SVM analysis helped reveal a more nuanced correlation between age, length of hospital stay, and OMSI severity, suggesting that these variables interact in a nonlinear manner to influence patient outcomes. Moreover, SVM provided additional predictive power by detecting subtle variable interactions that were not statistically significant in logistic regression but contributed to overall classification performance. This complementary approach strengthens the study’s findings by integrating both statistical and machine learning methodologies for a more comprehensive analysis of OMSI severity.

One limitation of our study is its retrospective design, which inherently limits the ability to establish causal relationships between risk factors and OMSI severity. Therefore, the findings should be interpreted with caution, as they reflect associations rather than definitive cause-and-effect relationships. Furthermore, as a single-center study, the results may not be generalizable to other healthcare settings with different patient populations and treatment protocols. These factors highlight the need for prospective, multi-center investigations to validate our results and improve external validity. In particular, future studies should explore the impact of non-odontogenic infections, assess the differential outcomes of surgical versus non-surgical treatment strategies, and adopt a longitudinal design to better understand disease progression and treatment effects over time. Comparative analyses of surgical and non-surgical approaches—considering treatment efficacy, cost-effectiveness, and patient-reported outcomes—will help optimize OMSI management based on disease severity and overall patient condition.

In addition to conventional epidemiological and clinical analyses, emerging technologies such as oral and maxillofacial organoid models offer promising opportunities to investigate OMSI pathophysiology and to develop personalized treatment strategies. Organoid technology enables in vitro replication of key disease processes, providing a platform for preclinical drug testing and mechanistic exploration, although technical and biological challenges remain [27]. Furthermore, epidemiological variations should be considered when interpreting and generalizing findings. For instance, a systematic review by Pereira et al. (2022) highlighted that road traffic accidents and assaults are leading causes of oral and maxillofacial trauma in certain regions, and these injury patterns have been associated with OMSI severity [28]. Future multi-center studies should integrate such population-level differences to refine prevention and management strategies.

## Conclusion

The severity of oral and maxillofacial space infection is associated with age, trismus, dysphagia, dyspnea, surgery, and length of hospital stay. While these factors are correlated with OMSI severity, it is important to note that due to the cross-sectional nature of our study, we cannot establish causal relationships. Further longitudinal studies are needed to confirm these associations and explore their causal mechanisms. Based on our findings, clinicians should prioritize early detection and management of key risk factors, such as trismus and dysphagia, to reduce complications and improve outcomes. Tailored treatment strategies, including a combination of antibiotic therapy and timely surgical intervention, should be considered for patients with severe OMSI. The findings of this study provide new insights into the diagnosis, treatment, and rehabilitation of oral and maxillofacial space infection, helping to better formulate and adjust treatment plans, improve patient prognosis, enhance patients’ quality of life, and contribute to the future development of the medical industry.

## Data Availability

The datasets generated during the current study are not publicly available due to patient privacy restrictions, but are available from the corresponding author on reasonable request.

## Abbreviations

OMSI: Oral and Maxillofacial Space Infection
SVM: Support Vector Machine
OR: Odds ratios
CI: Confidence intervals
